# Contribution of Visuospatial and Motion-Tracking to Invisible Motion

**DOI:** 10.3389/fpsyg.2016.01369

**Published:** 2016-09-14

**Authors:** Luca Battaglini, Clara Casco

**Affiliations:** Department of General Psychology, Perception, and Psychophysics, University of PadovaPadova, Italy

**Keywords:** motion extrapolation, invisible motion, motion-tracking, visuospatial attention, spatial cue

## Abstract

People experience an object's motion even when it is occluded. We investigate the processing of invisible motion in three experiments. Observers saw a moving circle passing behind an invisible, irregular hendecagonal polygon and had to respond as quickly as possible when the target had “just reappeared” from behind the occluder. Without explicit cues allowing the end of each of the eight hidden trajectories to be predicted (length ranging between 4.7 and 5 deg), we found as expected, if visuospatial attention was involved, anticipation errors, providing that information on pre-occluder motion was available. This indicates that the observers, rather than simply responding when they saw the target, tended to anticipate its reappearance (Experiment 1). The new finding is that, with a fixation mark indicating the center of the invisible trajectory, a linear relationship between the physical and judged occlusion duration is found, but not without it (Experiment 2) or with a fixation mark varying in position from trial to trial (Experiment 3). We interpret the role of central fixation in the differences in distinguishing trajectories smaller than 0.3 deg, by suggesting that it reflects spatiotemporal computation and motion-tracking. These two mechanisms allow visual imagery to form of the point symmetrical to that of the disappearance, with respect to fixation, and then for the occluded moving target to be tracked up to this point.

## Introduction

The visual experience of motion elicited by an object moving behind a stationary occluder has often attracted the attention of psychologists because of the paradoxical fact that the object persists in being “seen” as continuously moving behind the occluder through time, even though it is no longer projected onto the retina. One of the first demonstrations of occluded (“invisible”) motion is given by Michotte (Michotte et al., 1964, 1991). Within this acceptation, invisible motion is another example of a motion phenomenon that involves the subjective impression of an object following a path even in the absence of any physical stimulus, such as during apparent motion (Wertheimer, 1912). Within this framework are the studies that conceive invisible motion as equivalent to an amodal filling-in and as involving neural activation to visible motion (Michotte et al., 1964, 1991; Pessoa and Neumann, 1998; Horowitz et al., 2006; Komatsu, 2006). Empirical evidence comes from the finding that distractors moving over the occluder interfere with invisible motion (Lyon and Waag, 1995). At the neurophysiological level, Barborica and Ferrera (2003) have provided direct evidence of the existence of velocity sensitive neurons in the frontal eye fields that fire during periods of occlusion.

A different and very accredited model for processing occluded motion investigated by DeLucia and Liddell (1998) and expanded upon by Makin and Poliakoff (2011) regards the tracking hypothesis. They claim that the position of a hidden moving object is “extrapolated” by tracking the position of the target through the shift of the spotlight of visuospatial attention, which is guided by the motion pursuit system. Furthermore, they posit that, when the target disappears, visible velocity information stored in short-term velocity memory guides pursuit eye movements across the temporal intervals during which the target is occluded (Bennett and Barnes, 2006; Makin and Chauhan, 2014). Indeed, invisible motion is affected by factors affecting perceived visible speed before occlusion such as, for example, changes in the target's contrast, size (Battaglini et al., 2013), prior adaptation (Gilden et al., 1995; Battaglini et al., 2015) and previously viewed velocity (Makin et al., 2008). In Makin and Poliakoff's model, it is irrelevant whether the eyes follow the hidden moving object or not, thus absorbing into the model the evidence that premotor pursuit commands do not need pursuit execution to be active (Rizzolatti et al., 1994; Barnes et al., 1997; Eimer et al., 2007). In its complete account, the model posits that “*velocity store and premotor modules guide tracking of occluded targets during motion extrapolation, even if fixation is maintained”* (Makin and Poliakoff, 2011).

From this account, visuospatial attention seems to rely exclusively on the memory of visible motion. However, in particular, the results of Lyon and Waag (1995) and Barborica and Ferrera (2003) suggest that motion information that is also acquired during the occluded trajectory may be used to judge target reappearance. If this were the case, then the imagery of an occluded target in motion could guide pursuit eye movements across the temporal intervals during which the target is occluded (Lu and Sperling, 1990; Sears and Pylyshyn, 2000; Shioiri et al., 2000; Huber and Krist, 2004; de'Sperati and Deubel, 2006; Jonikaitis et al., 2009). The internal model of the moving target can be tracked smoothly, even though the target is not physically present, allowing the target position to be updated very precisely at every (very close) local image point along the occluded trajectory (Shioiri et al., 2000). Shioiri et al. (2000) indeed showed that observers judge the apparent location of a target in invisible motion relative to an imaginary cue with high precision, suggesting that the target motion behind the occluder can be tracked and that any position of the target along the occluded trajectory can be precisely judged, providing that this point is made salient by visual imagery.

Spatiotemporal computation is needed to form an internal representation of a moving object. Thus, rather than using remembered speed to track one speed dimension (location) to judge the other (time), motion-tracking uses remembered speed to track the two dimensions combined (motion) and to infer time (Cavanagh, 1992; Verstraten et al., 2000; Shioiri et al., 2002). Rather than exploiting information achieved by spatial filtering, motion tracking exploits information provided by spatiotemporal filters, i.e., filters devoted to spatiotemporal computation underlying the coding of speed by the motion system (see Burr and Thompson, 2011; Mather et al., 2012, for a review). Doherty et al. (2005) showed that when pre-occluder motion generated expectations concerning the where and when of reappearance, reaction times to reappearances are shortened, especially when spatial and temporal expectations combine. These differences may reflect a difference with respect to the way covert-attention is deployed during occlusion: attention directed to space and time combined (motion) may be more efficient than visuospatial attention directed to space alone.

**Figure 1:**
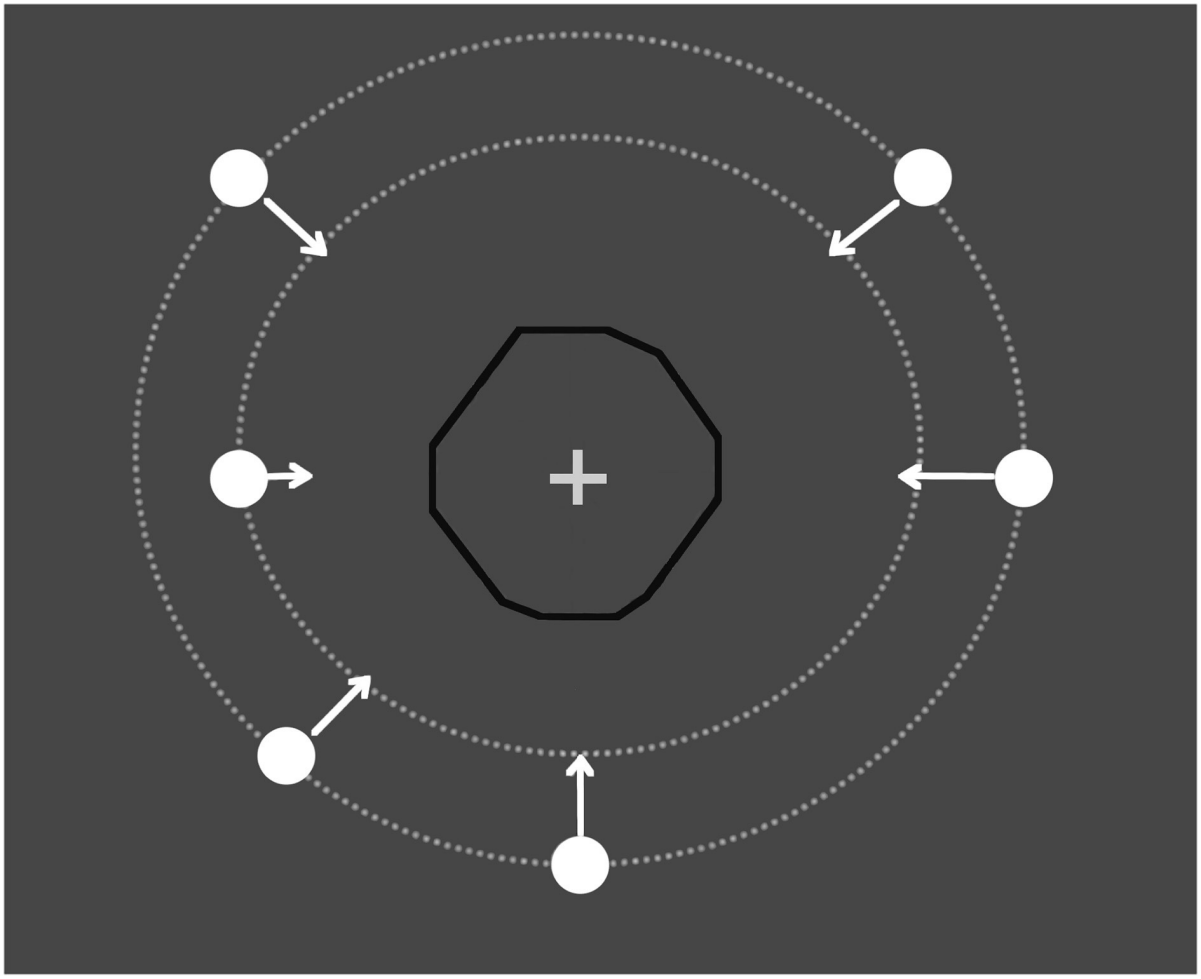
**Illustration of the trial**. A moving circle traveled through an invisible occluder (the black line is shown in the figure only for illustrative purposes) with an irregular polygon shape. The target (circle) started from eight different places at one of two different distances from the occluder. The participants had to press a response button as soon as the target reappeared. The RT was the interval between the key press and when the leading edge of the target reached the edge of the invisible occluder.

To assess the role of motion tracking we need to demonstrate that the time of arrival is judged on the basis of space and time combined, rather than on the computation of a separate motion dimension—either space or time. To this end, we made the occluder invisible and its shape unpredictable (as Figure 1 shows, it was an irregular hendecagonal polygon with bilateral symmetry in all directions), and abolished the reappearance cue that is typically used in experiments on motion extrapolation. In these conditions, spatiotemporal computation was precluded and observers were forced to respond either when they actually saw the target reappear or when they predicted its reappearance by “learning” the average trajectory length (spatial cue) or the average duration of occlusion (time cue). However, by placing a spatial cue centered on the invisible occluder we created the conditions for spatiotemporal computation. Indeed, occlusion duration can be combined with trajectory length (from disappearance to the cue centered on the occluder) to judge precisely when the target reaches the central cue. Assuming the lengths of the trajectory before and after the central cue are equal, reappearance can be “visualized” by imagery to allow spatiotemporal computation and motion pursuit from the central cue to reappearance. If the fixation mark is not central, motion tracking would never allow reappearance to be judged precisely. The same outcome is expected if the fixation mark is absent.

To establish the role of the spatiotemporal computation underlying motion tracking and evaluate its precision, we need evidence that anticipation errors also occur to guarantee that reappearance is anticipated. Most importantly, we need evidence of a linear relationship between the estimated time to reappearance (TTR_*estimated*_), calculated from the moment in which the target is in the center of the invisible occluder to the button press) and the actual duration of the half-trajectory length (TTR_*physical*_).

To sum up, predictions depend on whether stimulus conditions allow motion tracking or not:

- If the *visible speed, occluder shape (irregular and invisible), and reappearance point are unknown*, then observers cannot predict (anticipate) the target reappearance behind the occluder and are forced to respond when they actually see the target. We predict a linear relationship between TTR_*physical*_ and TTR_*estimated*_, with no anticipation errors.
- If the *visible speed is known but not the occluder shape (irregular and invisible), and there is no reappearance cue*, and the central cue is either absent or not central, then the exact reappearance point is unknowable. However, reappearance may be predicted, based on inferred unprecise occluder shape and using as a cue for predicting reappearance an average duration of the trajectories. In this case, anticipation errors may occur but TTR_*physical*_ and TTR_*estimated*_ are not positively related because the average trajectory length differs from individual trajectory lengths. Note that if an observer use an average strategy for judging the duration of occlusion we should obtain a flat slope when plotting 2 × TTR_*estimated*_ against 2 × TTR_*physical*_. However, since we considered (see Analysis Section) on the y axis the duration estimated from the center of occlusion, we removed also of the entire physical duration on the x axis that (obviously) is different according to the different trajectory lengths: smaller for a short trajectory and larger for a long trajectory. This way, when plotting TTR_*estimate*_ against TTR_*physical*_ we should obtain a negative slope when people predict target reappearance using an average value of the occlusion lengths. Moreover, to confirm that observers estimate an average duration of occlusion from the different trajectory lengths, a linear relationship between the RT (TTR_*estimated*_- TTR_*physical*_) and the TTR_*physical*_ with a negative slope is also expected.
- If the *visible speed is known but not the occluder shape (irregular and invisible) and there is no reappearance cue*, but there is a visible cue centered on the occluder, then this may allow a spatiotemporal computation and the formation of an internal representation of the occluded moving target so that it can be “tracked” during its trajectory from disappearance to the central cue and from there to reappearance, “visualized” as symmetrical to the disappearance with respect to fixation. In addition to anticipation errors, a linear relationship between TTR_*physical*_ and TTR_*estimated*_ is expected. Thus, the crucial finding to infer that motion tracking has occurred, based on spatiotemporal computation, is the linear relationship between TTR_*estimated*_ and physical duration.

## Experiment 1

Experiment 1 aims to disentangle outcome (a) from outcomes (b) and (c). Whereas pre-occluder motion allows participants to anticipate the target reappearance, this is impossible without pre-occluder motion, and observers can only respond when they see the target. That is, in this second baseline condition we do not predict anticipation errors without pre-occluder motion, whereas TTR_*estimated*_ should depend on trajectory length.

### Methods

#### Participants

Seven students from the University of Padova (4 female, 3 male; age 19–22 years) participated voluntarily in Experiment 1. The participants remained unaware of the true aims of the experiment until they completed the task. All of the participants gave written informed consent in accordance with the Declaration of Helsinki.

#### Stimuli, apparatus, and procedure

The participants were placed in a dark room, seated 57 cm away from the display screen. The viewing was monocular, and both eyes were tested. Stimuli were generated with Matlab Psychtoolbox (Brainard, 1997; Pelli, 1997) and displayed on a 19-inch Asus monitor with a refresh rate of 60 Hz. The screen resolution was 1920 × 1080 pixels. Each pixel was subtended ~1.5 arcmin. The luminance of the background was 0.7 cd/m^2^. The target was a small circle that was 0.5 degree of visual angle (deg) in diameter whose motion remained invisible when the disk passed behind an invisible irregular hendecagonal polygon. A fixation cross 0.3 deg long and 0.1 deg wide (60 cd/m^2^) was placed in the center of the occluder. Both had a luminance (as measured by a Minolta LS−100 photometer) of 90 cd/m^2^. In one block, the target initiated a linear trajectory after a randomly chosen interval of 0–2000 ms from an acoustic cue either 7.5 or 10 deg from the center of the screen and terminated 4 deg after reappearance. In the other block, the visible pre-occluder trajectory was removed and the target motion started from the center of the occluder (the target was invisible behind the occluder). In this block, the observers knew where but not when the hidden trajectory started. The target speed (either 3 or 6 deg/sec) was randomly selected within each block. The direction was randomly chosen within each block. In the condition with pre-occluder motion available, the trajectory could begin from either side of the screen, from one of eight specified directions, separated by a 45 deg sector of a virtual circumference: 0−180 (horizontal), 45–225 (diagonal, from upper-right to lower-left and vice versa), 90–270 (vertical), and 135–315 deg (diagonal from upper-left to lower-right and vice versa). Because the polygon is irregular, the hidden trajectory had a different length for each direction (Figure 1): (0−180: 5 deg; 90–270: 4.9 deg; 45–225: 4.75 deg; 135–315: 4.7 deg). Each block consisted of 64 trials: 2 repetitions of each direction, speed and starting position (7.5 or 10 deg). In all of the blocks, the participants were required to fixate on the central cross. A chin-rest was used to limit head movement.

The participants' task was to respond as quickly as possible when the target “just reappeared.”

#### Analysis

The physical time to reappearance (TTR_*physical*_)for each of the four trajectory lengths of 4.7, 4.8, 4.9, and 5 deg corresponded to 783, 800, 816, and 833 ms with a low-speed target and 391, 400, 408, and 417 ms with high speed, respectively [TTR_*physical*_: (invisible trajectory length/2)/speed of the target. TTR_*physical*_ was calculated from the center of the occluder because in one block of Experiment 1 the target started from the center]. We considered three dependent variables: (a) estimated TTR (TTR_*estimated*_), which corresponded to the response time measured from the center of the occluder to key press: TTR_*estimated*_ = TTR_*physical*_ + RT. (b) RT that is equal to the estimation of the entire duration of occlusion minus the entire physical duration of occlusion, corresponding to: (TTR_*estimated*_ + TTR_*physical*_) − 2 × TTR_*physical*_, i.e., half of duration estimated (that include the entire RT plus half of the physical duration) minus the entire physical duration of occlusion. The result is equal to TTR_*estimated*_ − TTR_*physical*_. (c) anticipation errors (negative RTs). Individual regression lines were fitted to evaluate the relationship between TTR_*physical*_ and TTR_*estimated*_, and between the RT and the TTR_*physical*_. We used either *t*-tests or ANOVA to compare the individual slopes obtained in the condition with fixed central cue with those obtained in the control condition of each experiment.

### Results

**Figure 2:**
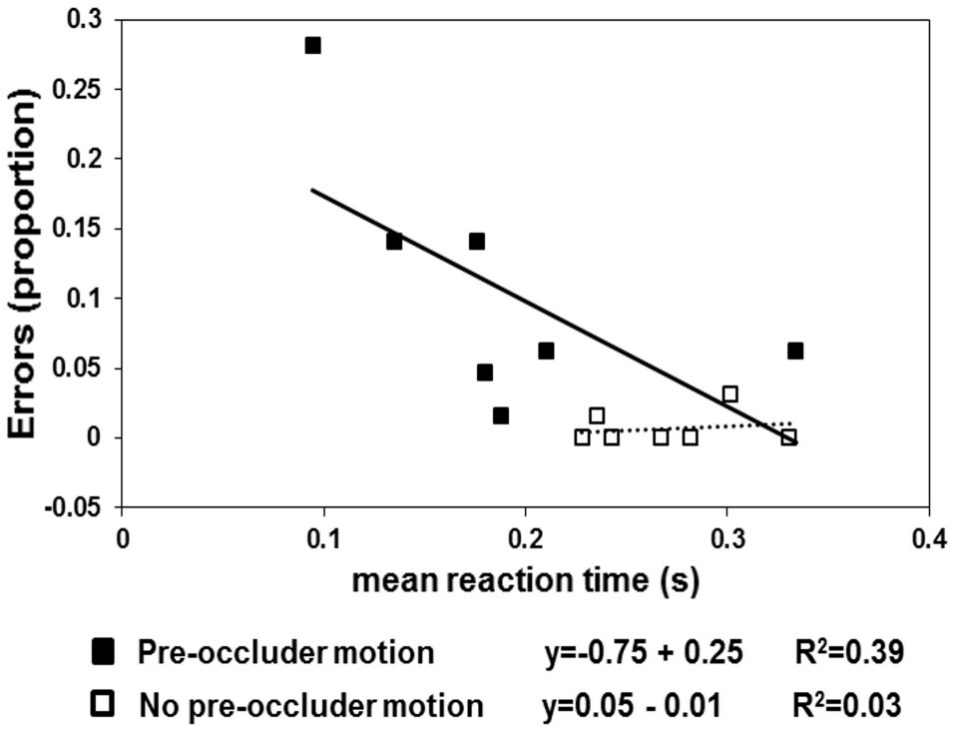
**The squares represent the proportion of errors (negative RTs) as a function of mean individuals (*n* = 7)**. The filled squares refer to the “pre-occluder” motion condition, and the empty squares represent the “no pre-occluder motion” condition. The linear regression lines are fitted to the “no pre-occluder motion” data (dotted line) and to the “pre-occluder motion” data (continuous lines).

**Figure 3:**
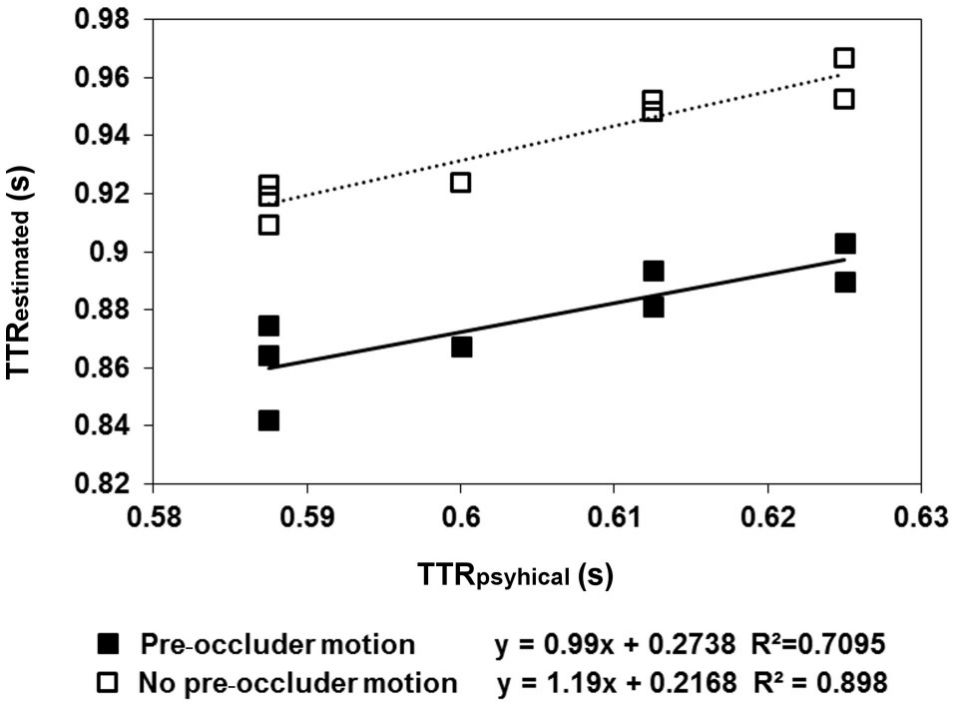
**The regression lines model the linear relationship between the average estimated time-to-reappearance (TTR_*estimated*_) data obtained in Experiment 1 and TTR_*physical*_**. The filled squares refer to the “pre-occluder motion” condition, whereas the empty squares refer to the “no pre-occluder motion.” The moving dot traveled along one of eight specified directions (four axes: 0–180, 90–270, 45–225, and 135–315°). The semi-axes are symmetrical with respect to the central fixation, except for the direction: 45–225, where a small asymmetry of the figure (0.5 mm) made the length of the trajectory 2.4 deg in the direction 45–225 and 2.35 deg in the direction 225–45. In the “pre-occluder motion” condition, the relationship between TTR_*estimated*_ and TTR_*estimated*_ was linear, as it was in the baseline condition, in which the participants were forced to respond when they saw the target reappearing. This reflects the distinction between individual trajectory lengths rather than response to average length.

The results are shown in Figures 2, 3. In the pre-occluder motion condition, there were more individual anticipatory errors, which were inversely related in a linear way to individual mean RTs (Figure 2). Figure 2 shows also that the individual mean RT are shorter with pre than without pre-occluder motion, indicating that short RT can be another measure of the tendency of the participants to anticipate target reappearance. Most importantly, in both conditions, TTR_*estimated*_ was directly related to TTR_*physical*_, indicating an isomorphic relationship between these two variables, a result implying that trajectory length/duration was judged with high precision (Figure 3).

One-sample *t*-tests revealed that the anticipation errors (negative RTs) differed from 0 (no errors) in the condition in which the pre-occluder trajectory was present [*t*_(6)_ = 3.151; *p* < 0.02] but not when it was absent [*t*_(6)_ = 1.14; *p* < 0.2]. Moreover, the regression lines fitted to the anticipation errors obtained as a function of RTs revealed a significant negative slope in the pre-occluder motion condition (slope = −0.75, *R*^2^ = 0.4) but not when the pre-occluder motion condition was absent (slope = 0.05, *R*^2^ = 0.03).

The average TTR data showed a linear relationship between TTR_*estimated*_ and TTR_*physical*_, both in the condition with pre-occluder motion (slope = 0.99 and *R*^2^ = 0.71) and in the baseline condition, without pre-occluder motion (slope = 1.19, *R*^2^ = 0.90). A *t*-test executed to evaluate the difference between the individual slopes obtained with pre-occluded motion either present or absent was not significant [*t*_(6)_ = 1.5; *p* = 0.17]. The results demonstrate that without pre-occluder motion, the observers responded when they saw the target. With pre-occluder motion present, the observers anticipated the target reappearance, and the evidence that TTR_*estimated*_ was isomorphic to TTR_*physical*_ indicated that they do so by a very precise spatiotemporal computation.

## Experiment 2

We ran a second experiment to confirm the hypothesis that, whereas anticipation errors may result from a computation of average trajectory length, the linear relationship between physical and judged trajectory duration does not. Shioiri et al. (2000) have shown that participants can precisely judge the apparent location of a target in invisible motion relative to an imaginary cue. We asked whether the participants could exploit this ability to judge target reappearance. They could “track” the target's motion from disappearance to when it reached the position behind the occluder marked by a visible cue (the central fixation) and then, by symmetry, from there to when it reached an imaginary cue signaling the point of reappearance, positioned symmetrically to the point of disappearance with respect to the central fixation (Figure 1). To test this possibility in Experiment 2, we compared the condition in which the cue indicating the center of the trajectory was available, thus allowing spatiotemporal computation, with the condition in which it was absent. In the first case, participants could “follow” the moving target behind the occluder for the first part of its trajectory up to when it reached fixation; for the second part, its length was isomorphic to the first, so visual imagery of the reappearance point was then available by motion-tracking. Conversely, when there was no cue and the trajectory length was not constant, the participants were either obliged to respond when they saw the target reappearing or to learn an average trajectory length or occlusion duration. Two groups were tested: the first was instructed to maintain fixation at the central cue, while the second could follow the moving target with their eyes.

### Methods

#### Participants

Two groups of seven students (7 women, 7 male; age 21–25 years) participated in Experiment 2.

All of the participants gave written informed consent in accordance with the Declaration of Helsinki.

#### Stimuli, apparatus, and procedure

This experiment was a replication of Experiment 1 (in terms of stimuli, apparatus, and procedure), with the difference being that pre-occluder motion was present in both conditions. However, in one condition, we removed the spatial cue (fixation cross) that indicated the center of the invisible trajectory. To narrow this experiment, the starting position of the target was always 7.5 deg from the center of the occluder and only one eye (dominant) was tested. The 14 participants were divided in two subgroups of seven subjects each: one subgroup performed the task while fixating on the center of the hidden trajectory; the other did not have any instruction to fixate. In the first group, to ensure fixation without a central mark, a circle (1.5 deg; 120 cd/m^2^) was placed over the blind spot (the participants were instructed that for correct fixation to occur, the circle should remain not visible); in the other condition, the central fixation was present. Although blind spot is an imperfect method for detecting small saccades, it helps observers to follow the instruction of maintaining central fixation rather than following with the eyes the hidden moving target.

### Results

**Figure 4:**
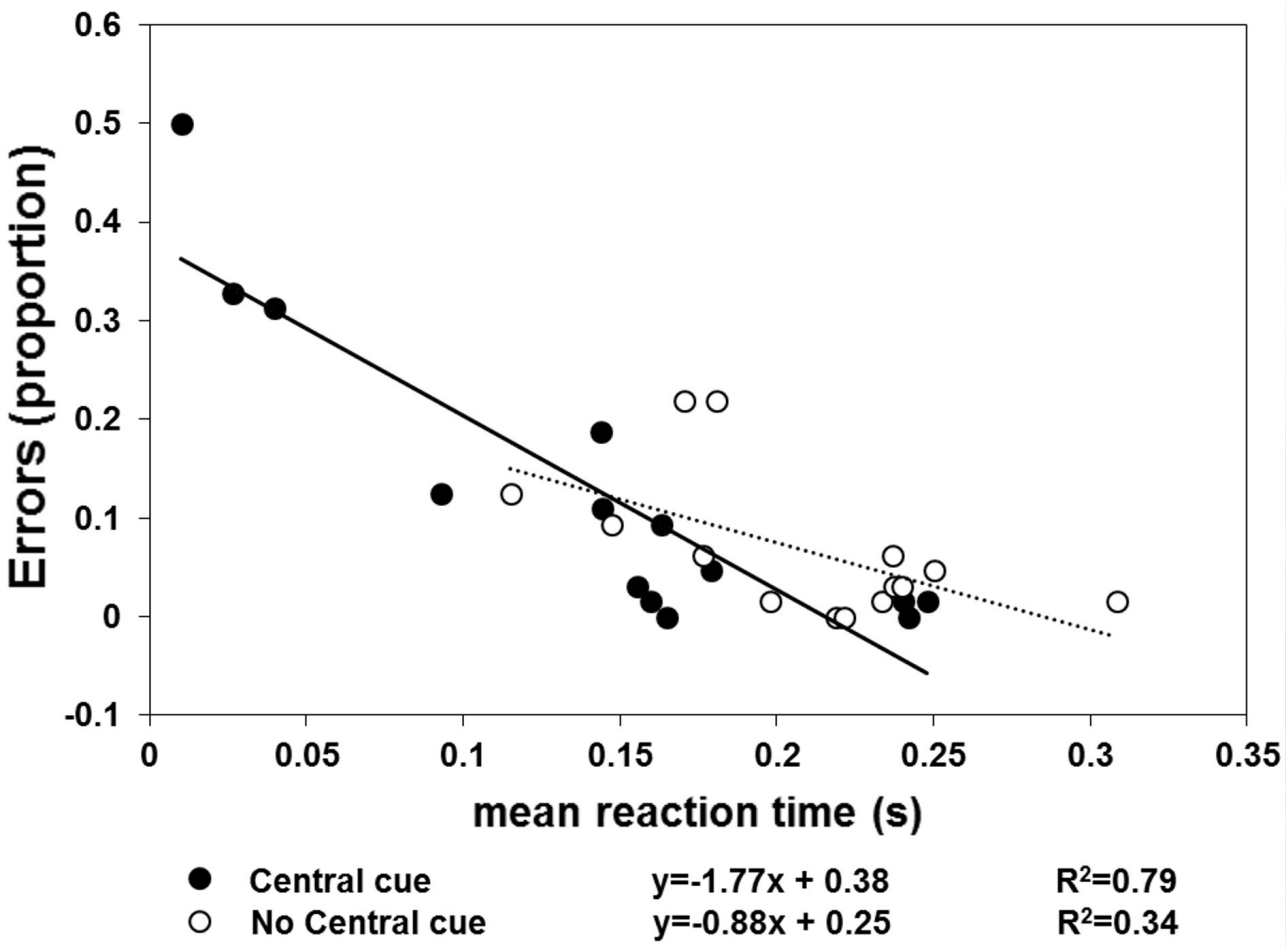
**Individual proportions of errors (*n* = 14) are plotted as a function of mean RT**. The filled symbols and continuous line refer to the “central cue” condition, whereas the empty symbols and dotted line represent the “no central cue” condition's results.

**Figure 5:**
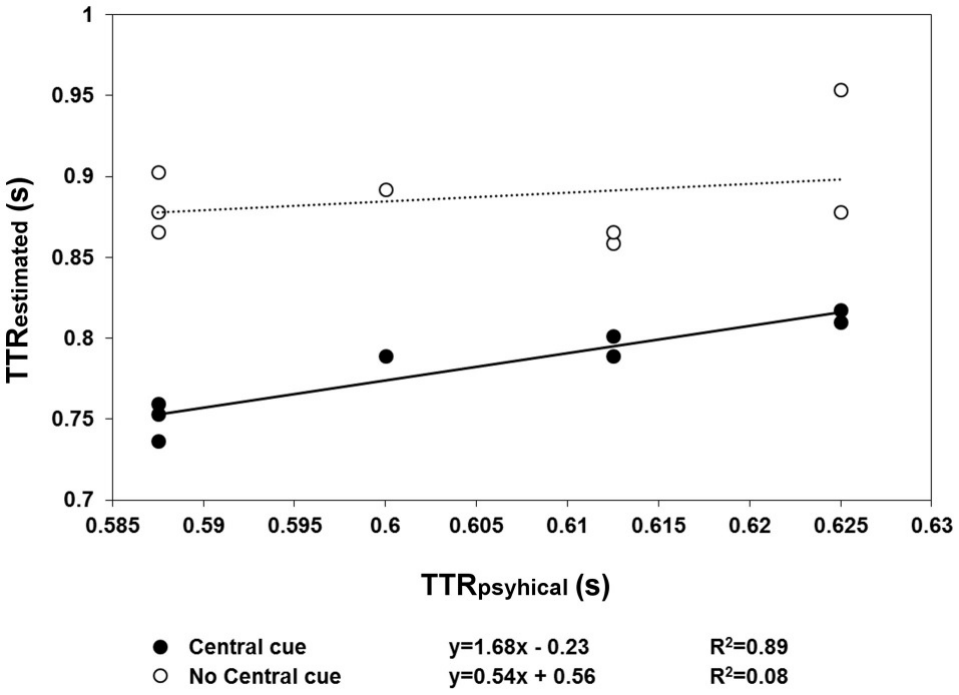
**The mean TTR_*estimated*_ data averaged across speed are plotted as a function of TTR_*physical*_, separately for the “central cue” (filled symbols) and “no central cue” conditions (empty symbols)**. The regression lines are fitted to the “central cue” (continuous lines) and “no central cue” conditions (dotted line). Only in the condition with a central cue did the regression line (continuous line) reflect a linear relationship between TTR_*estimated*_ and TTR_*physical*_, indicating a temporal distinction between the individual trajectory lengths, rather than response to average length.

The results of Experiment 2 are shown in Figures 4, 5. With respect to when the central cue was absent, its presence produced a larger number of anticipatory errors, which was inversely related to RTs (Figure 4). Moreover, TTR_*estimated*_ was only isomorphic to TTR_*physical*_ with the central cue (Figure 5). Moreover, the relationship between RTs and TTR_*physical*_ were not linearly related with fixation present (suggesting that TTR_*estimated*_ but not RTs depend on the duration of occlusion: slope = 0.2, *R*^2^ = 0.09) and there is a weak linear (negative) relationship when the fixation was absent (slope = −0.75, *R*^2^ = 0.24). The mixed-design ANOVA on the number of errors having group and central fixation (present vs. absent) as factors revealed that the effect of group was not significant [*F*_(1, 12)_ = 0.42, *p* = 0.53, η^2^_*p*_ = 0.34], while the effect of central fixation was significant [*F*_(1, 12)_ = 4.75, *p* = 0.049, η^2^_*p*_ = 0.28], indicating that the number of errors (Figure 4) was higher in the central cue condition [*t*_(13)_ = 2.23, *p* = 0.04]. The slope of the regression line fitted to the errors plotted as a function of the RTs indicated a larger slope with the central cue present (slope = −1.77, *R*^2^ = 0.79) than absent (slope = −0.88, *R*^2^ = 0.34). Most importantly, the relationship between the physical and average TTR_*estimated*_ (Figure 5) was linearly positive when the central cue was present (slope = 1.68, *R*^2^ = 0.89) but not when absent (slope = 0.56, *R*^2^ = 0.08). The ANOVA executed to evaluated the difference between the individual slopes in the two cue conditions (present vs. absent) revealed a significant effect of group (*p* = 0.01) and condition [*F*_(1, 12)_ = 4.74, *p* = 0.049, η^2^_*p*_ = 0.28], indicating higher slopes with cue present and higher slopes in the group that did not receive instructions to fixate.

This suggests that with and without a central mark, the judgment of target reappearance may be based on different information. Under the assumption that a linear positive relationship between TTR_*physical*_ and TTR_*estimated*_ reflects motion tracking, mediated by spatiotemporal computation during occlusion, this information is only available with the central cue. In the absence of a central cue, the anticipation of reappearance may rely on a “learned” average trajectory length/duration. However, this would produce negative slopes when TTR_*estimated*_ are plotted as a function of TTR_*physical*_ and when RTs are plotted as a function of TTR_*physical*_. It was not found any strong or medium correlation, therefore it is unlikely that observers use as a cue for predicting target reappearance an average duration of occlusion when the fixation cross is not present. Furthermore, the fixation strategy does not affect qualitatively the effect due to the presence of the central cross, although the individual slopes were steeper in the subgroup in which fixation was not needed. This suggests that the information coming from the oculomotor system can improve accuracy but does not affect the isomorphic relationship between TTR_*estimated*_ and TTR_*physical*_.

## Experiment 3

In the last experiment, we further sought to confirm the role of spatiotemporal computation in judging reappearance. This was done, as in Experiment 2, by evaluating the role of the central visible cue to “visualize” the point of reappearance, positioned symmetrically to the point of disappearance with respect to the central cue (Figure 3).

To this end, we replicated the conditions of Experiment 2 (same stimulus, apparatus, and procedure) with the central cue available and compared it with a new condition, in which we randomly varied the position of the central cue from trial to trial, either to the left or to the right with respect to the center. In this second case, the lengths of the two half-trajectories were not equal in most trials, so the central cue could not be used to correctly infer the target reappearance. Therefore, the participants could either respond when they saw the target reappear or “learn” the average occlusion duration of the invisible trajectory by forming a visual representation of the occluder shape. The two conditions are presented in separate blocks.

### Methods

#### Participants

Twelve students (6 women, 6 males; age 21–33 years) from the University of Padova participated in this experiment. All of the participants gave written informed consent, in accordance with the Declaration of Helsinki.

#### Stimuli, apparatus, and procedure

In this experiment, we replicated the stimuli, apparatus, and procedure used in Experiment 2 with the following differences: in one of the two blocks, presented in counterbalanced order, the visible cue was positioned centrally or either behind or ahead with respect to the center (3 levels) of the occluder at a distance of 0.3 deg from it (variable fixation condition), whereas in the other block, the central cue was fixed (fixed condition) at the center of the invisible trajectory. The participants performed 96 trials in each block (in the first one, there were 2 repetitions × 8 target directions × 3 fixation conditions × 2 speeds; in the second block, there were 4 repetitions × 8 target directions × 1 fixation condition × 2 speeds). The viewing was binocular, and the participants were requested to fixate on the visible cue.

### Results

**Figure 6:**
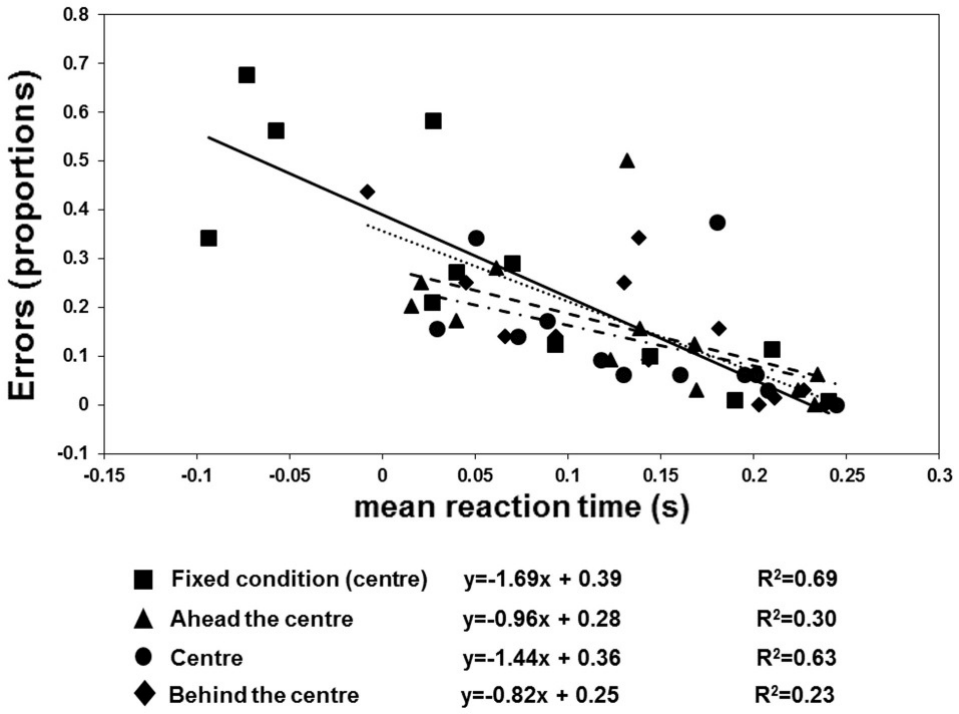
**Individual proportion of errors (negative RTs) as a function of mean RT, fitted by regression lines**. The squares plus continuous line refer to the “fixed condition.” The three variable-fixation conditions are represented by triangles plus broken lines (fixation ahead the center), diamonds plus dotted lines (fixation behind the center), and circles and broken dotted lines (fixation central), respectively.

**Figure 7:**
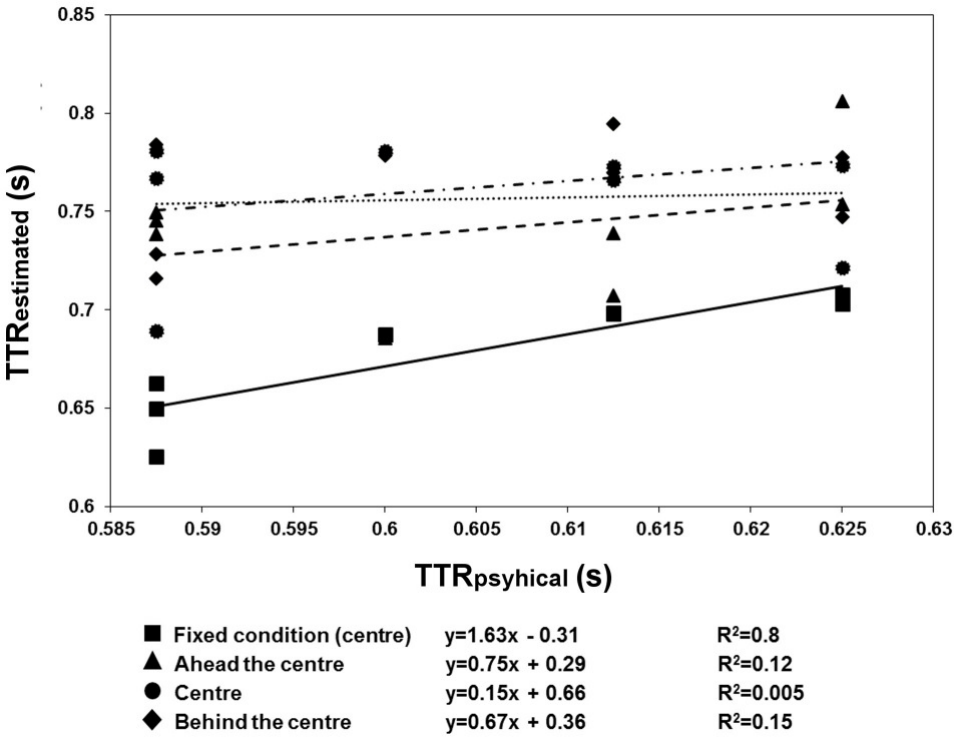
**Regression line fitted to the mean TTR_*estimated*_ data of Experiment 3, averaged across speed**. Fixed fixation data: squares plus continuous line; variable fixation data: triangles plus broken lines (fixation ahead), diamonds plus dotted lines (fixation behind), and circles plus broken dotted lines (fixation central). In the fixed condition, the relationship between TTR_*estimated*_ and aTTR_*physical*_ was linear, indicating a temporal distinction between individual trajectory lengths rather than response to average length.

The results are shown in Figures 6, 7. There were more anticipatory errors in the fixed fixation condition. With a central cue (both in the fixed and variable conditions), there was a linear, negative relationship between the errors and RTs (Figure 6). Moreover, a linear positive relation between TTR_*estimated*_ and TTR_*physical*_ was only found in the fixed condition (Figure 7).

The anticipation errors were analyzed with a repeated-measures ANOVA with the condition (variable: behind, ahead, and central vs. fixed cue) and speed of the target (3 vs. 6 deg/sec) as factors. The results reveal that the anticipatory errors were affected by speed [*F*_(1, 11)_ = 5.79, *p* = 0.035, η^2^_*p*_ = 0.35] and condition [*F*_(1.097, 12.066)_ = 16.62, *p* = 0.001, η^2^_*p*_ = 0.6]. *Post-hoc t*-tests with Bonferroni correction revealed that the number of errors was greater with a fixed than with a variable position of the central cue (fixed vs. behind: *p* = 0.01; fixed vs. central: *p* = 0.008; fixed vs. ahead: *p* = 0.012). However, the RTs and errors were linearly related in both conditions with the central cue (fixed condition: slope = −1.69, *R*^2^ = 0.69; variable condition: slope = −1.44, *R*^2^ = 0.63). Most importantly, the analysis on TTRs revealed that the relationship between TTR_*physical*_ and average TTR_*estimated*_ was linear positive in the fixed condition (slope = 1.63, *R*^2^ = 0.8) but not in the variable one (ahead: slope = 0.75, *R*^2^ = 0.12; central: slope = 0.15, *R*^2^ = 0.005; behind: slope = 0.67, *R*^2^ = 0.15). The ANOVA executed to evaluate the differences between the individual slopes in the four different cue conditions (fixed central, variable ahead, variable central, and variable behind) revealed a significant effect of condition [*F*_(3, 33)_ = 4.9, *p* = 0.006, η^2^_*p*_ = 0.31]. *Post-hoc* Bonferroni corrected *t*-tests indicated higher slopes with the fixed central cue than with the cue having a variable position: ahead, *p* = 0.022; central, *p* = 0.049; behind, *p* = 0.047). The flat slopes obtained when the cue is in variable positions suggest that participants did not use an average value of occlusion to predict target reappearance. Moreover, it was tested whether RT are inversely associated with the TTR_*physical*_ when the cue is not fixed and it was found very weak correlation for each condition (ahead: slope = −0.33, *R*^2^ = 0.04; center: slope = −0.84, *R*^2^ = 0.15; behind: slope = −0.25, *R*^2^ = 0.01) confirming that is unlikely that participants use an average value to estimate target reappearance.

Note that, since fixation was available in both conditions, the effect of condition found in Experiment 3 cannot be accounted for by different fixation strategies in the two conditions (a possible confounding variable of Experiment 2, even though this confounding variable should have been eliminated or limited by allowing free eye movement in one subgroup).

## Discussion

We used a new paradigm to investigate invisible motion. We abolished any info on occluder size and shape, and abolished any cue that could signal when and where the target would reappear. We asked whether the observers would anticipate reappearance and produce a time-to-reappearance (TTR_*estimated*_) isomorphic to the TTR_*physical*_, whose duration varied randomly from trial to trial. Anticipation errors were found in all conditions except when information on pre-occluded motion was not available, in the baseline condition of Experiment 1 (Figure 2). Regarding the relationship between TTR_*physical*_ and TTR_*estimated*_, Experiment 1 shows that it was linear (positive) with pre-occluder motion as well as without, when the participants were forced to respond when they saw the target (Figure 3). Experiment 2 showed a linear positive relationship between these two variables when a cue indicating the middle of the hidden trajectory was present, but not when it was absent. In Experiment 3, the linear positive relationship between TTR_*physical*_ and TTR_*estimated*_ was only found when the position of the central cue was fixed, and not when it varied randomly within the block. These results support the spatiotemporal computation hypothesis: to judge trajectory length and anticipate reappearance, participants must first judge when the target reaches the position behind the occluder marked by the central fixation and then, by symmetry, when it reappears in an opposite symmetrical position relative to the point of disappearance. We suggest that spatiotemporal computation allows motion tracking and a very precise visual imagery of the point of reappearance. In sum:

- *When the observers do not have visible motion available* as in the baseline condition of Experiment 1, in which the target appears from behind an occluder without knowing where and when its trajectory started, the participants respond when they actually see the target; the response is a true reaction time without anticipation errors and reflects a linear relationship between TTR_*physical*_ and TTR_*estimated*_.
- *When the fixation mark is either absent or not fixed in the center of the hidden trajectory*, anticipation errors may occur but the relationship between TTR_*physical*_ and TTR_*estimated*_ is not linear. This result suggests that although the point of reappearance is unknown, observers predict—not precisely—the target reappearance. One hypothesis is that observers can implement a strategy in which they estimate an average duration of occlusion from the different trajectories length. However, the absence of negative slopes in the linear regressions obtained when plotting TTR_*estimated*_ against TTR_*physical*_ and the RT against the TTR_*physical*_ did not support this hypothesis. Furthermore, also subjective reports of the occluder shape does not support the previous hypothesis. Indeed, about half of the observers reported that the occluder was a circle and then a figure with equal trajectories lengths, but the other half reported that the occluder was an ellipse or a square, a figure with different trajectories lengths (however none of them describe the occluder as being a hendecagonal polygon). Another possible strategy is tracking the current spatial position of the target with the shift of the visuospatial attention (Makin and Poliakoff, 2011). Indeed, attention has been shown to be independent of the strength of the stimulus (Doherty et al., 2005; Boynton, 2009), and its effects have been seen in the absence of visual stimulation (Kastner et al., 1999; Murray, 2008) and to empty regions of space (Serences and Boynton, 2007). For simple attentive visuo-spatial tracking a central cue is not needed, and when it is present a saccade-like shift of attention may be favored (Cave and Bichot, 1999; Chastain, 1992a,b).
- *When there is a central fixation*, this leads to many anticipation errors (negative RTs) and to a linear positive relationship between TTR_*physical*_ and TTR_*estimated*_. Considering that the four values of trajectory lengths range between 4.7 and 5 deg and that the observers experience, for each speed, only 4 trials for each randomly presented trajectory, it is “impossible” to learn this difference so precisely to justify the linearity found between the dependent and independent variables. Obviously, the anticipation of target reappearance here involves more low-level computation mechanisms than spatial attention or memory. The crucial role of central fixation suggests that spatiotemporal computation behind the occluder occurs and that the output of spatiotemporal filtering mediates precise motion-tracking along the hidden trajectory. Because occluded motion prevents sensory input from reaching the visual system, we posit that visual imagery of the moving stimulus must be formed to extract motion information behind the occluder, so that the stimulus can be tracked from disappearance to fixation and then from fixation to a symmetrical position to disappearance. Based on motion tracking, reappearance can be judged almost as precisely as if the target were visible (Shioiri et al., 2000). Indeed, imagery is not very different from weak sensory stimulation, as both produce perceptual effects and accumulate over time (Raymond, 2000; Pearson and Brascamp, 2008).

Motion tracking of visual imagery during occluded motion share some similarities with amodal filling-in (amodal completion) (Ferree and Rand, 1912; Casco and Morgan, 1984; Ramachandran and Gregory, 1991; Ramachandran, 1993; Grassi and Casco, 2010; DeStefani et al., 2011), by which neural activation spreads at the point of reappearance or, retrospectively, from the imagined reappearance point (interpolation) (Hogendoorn et al., 2008). Both operations allow the use of a set of discrete spatial positions to form an internal model of the moving target. However, imagery-motion tracking is more likely to be mediated by feature-based attention, whereas filling-in does not necessarily involves attention (Komatsu, 2006).

Therefore, our results unveil the role of motion tracking during occluded motion. Indeed, previous studies using stimuli involving motion tracking found results compatible with ours (Shioiri et al., 2000). One similarity is the use of a set of discrete spatial positions to form an internal model of the moving target, which allows its motion to be tracked across intermediate spatial positions. In addition, motion tracking is linear, consistent with our results of an isomorphic relationship between TTR_*physical*_ and TTR_*estimated*_. Moreover, motion tracking produces location judgments, as it does for continuous motion. Therefore, it may well account for anticipation of the reappearance of the moving target. In addition, motion tracking occur at relatively long SOAs (Shioiri et al., 2000) and the duration of the invisible trajectory from disappearance to the position marked by central fixation is indeed ~800 ms at low speed. Finally, Shioiri et al. (2000) showed that the critical factor for motion tracking is SOA and not speed; indeed, in the present work, we found similar results at low and high speed (see Figure 7).

It is possible to argue that the location of reappearance could be used to predict the time of reappearance. Therefore, motion tracking, involving the visibility of objects to be tracked (Cavanagh, 1992) would not be strictly necessary. Time to reappearance from fixation could be predicted by simply waiting for the same duration as passed. However, our knowledge of how attention to moving objects works suggests spatiotemporal computation. Shioiri et al. (2002) showed that attention does not simply select a location for enhanced processing, but rather predicts the future location of the object of interest based on its velocity. Cavanagh (1992) showed that motion tracking provides accurate velocity judgments. Verstraten et al. (2000) showed that if temporal frequency is not too high (temporal limit is 4–8 Hz) tracking involves localization in both the spatial and temporal domain as motion tracking does. Moreover, there are at least two pieces of evidence supporting our claim that motion tracking (implicating spatiotemporal computation) is involved in the conditions with central cue. One is in the psychoacoustic domain. Matthews and Grondin (2012) showed that the Weber fraction for duration discrimination of paired of sound is around 4% when the baseline stimulus is presented for 1 second (40 ms). In our paradigm the minimum differences in duration of the entire invisible trajectory is 40 ms for a low-speed target (3 deg/s) and 20 ms for a high-speed target (6 deg/s). It is then highly unlikely that participants use a timing strategy rather than motion-tracking and this is even more unlikely knowing the higher temporal resolution to the auditory with respect to the motion system. More direct is the evidence that with visible cues of reappearance present and a subjective task, the judgements of reappearance are imprecise. For example, in DeLucia and Liddel1 (1998) the observers had to judge whether a target object reappeared in time or not. When the reappearance error was 0 the accuracies were around 70%.

In conclusion, the evidence presented in the present study is consistent with an active process underlying occluded motion that produces an internal spatiotemporal model of the moving target, mediated by high-resolution visual mechanisms (Koenig-Robert and VanRullen, 2011). We do not only emphasize attentional tracking, time processing, or visuospatial updates of the attention spotlight (Tresilian, 1995, 1999; Makin and Poliakoff, 2011; Makin and Chauhan, 2014). Instead, we want to highlight that the elaboration of occluded motion is an active process that, in appropriate conditions, is coupled with visual imagery. To our view, motion tracking does not substitute for but is additive to visuospatial tracking, in the sense that it only works in appropriate conditions. However, given its relevance, it should be incorporated into the models of motion extrapolation. Visuospatial tracking and motion-tracking are indeed complementary processes. Indeed, visuospatial attention selects spaces, whereas motion tracking may select the imagery of a visual dimension like direction of motion, speed, or motion path. Without denying the importance of shifting attention between different locations of the occluded target to track the target's location along its trajectory, the operation of tracking particular visual features of the invisible motion (speed, direction, or spatial-temporal frequency combined) may be the prerequisite to judge reappearance with high precision, not only experimentally but also in daily life.

## Author contributions

Study conception and design: LB, CC. Acquisition of data: LB. Analysis and interpretation of data: CC, LB. Drafting of manuscript: LB, CC. Critical revision: CC.

### Conflict of interest statement

The authors declare that the research was conducted in the absence of any commercial or financial relationships that could be construed as a potential conflict of interest.
